# Twenty-One or Twenty-Three?

**Published:** 1890-11-29

**Authors:** 


### The Hospital

A Weekly Journal of

Science, Medicine, Nursing, and Philanthropy.

*For Hospitals, Asylums, Infirmaries, Dispensaries, Medical Practitioners, Students, Nurses, and the Charitable Public.*

Vol. IX.—No. 218.] [November 29, 1890.

### TWENTY-ONE OR TWENTY-THREE?

No man can hold a curacy, even in deacon's orders, until he is twenty-three; he cannot hold a living until he takes priest's orders; and he cannot take priest's orders until he is twenty-four or more. Most people will consider that this is as it should be, and that if any change were thought necessary it would be in the direction of increasing the age which qualifies for ordination rather than of diminishing it. Such being the state of the case as regards clergymen, many persons who do not know will be surprised to learn that a man can acquire a licence to practice medicine at the youthful age of twenty-one; and not only so, but he is under no compulsion, as the man in deacon's orders is, to work under a senior for a time; but he can, even at that boyish age, dub himself doctor, put up his brass plate, and kill or cure as many people as choose to submit themselves to his care.

This is a question which the public should look into. It cannot be expected that the medical profession will be very anxious about it. True, it may be supposed that those doctors who are already in practice will desire to keep competitors out, and so will strive, in their own interests, to raise the licensing age. But on this point a very important fact is sometimes overlooked. It is this: that a large number of medical students are the sons of family doctors, and they are very much needed at home to help in the practice. Under these circumstances it is but natural that the father should wish the son to obtain his qualification at the earliest possible age. Not only does the elder thereby gain the much-needed relief of professional help in his daily work, but he also saves an outlay of one to two hundred a-year for college expenses. These are arguments which human nature finds it hard to resist.

The question, we say, is one for the public rather than for the medical profession; and the public may gain some definite ideas on the subject by comparing the youthful medical man with the newly-fledged curate. We shall not be thought unkindly critical if we say that the curate of twenty-three or twenty-four is seldom a man of very ripe wisdom; and still less is he a person of large and varied experience. But if the youthful curate of twenty-three or twenty-four is a comparatively inexperienced man, what must the young doctor be, who may be no more than twenty-one? To give the curate his due, he is generally modest, and is sometimes even painfully shy. There is, indeed, somewhere, a curate's letter in which the writer announces to a friend that his rector is going away for a six weeks' holiday, and that he will be "boss of the show," as he expresses it, for that period. But that curate is a young man of quite exceptional self-reliance and resource. The average deacon or newly-consecrated priest is a person who knows that he is young and that he has seen little of life, and he comports himself with that unassuming modesty which is so indicative at once of good sense and good feeling.

It cannot be said that modesty and a want of self-reliance are distinguishing characteristics of the youthful doctor. Although he may be years younger than the curate, he will not seldom behave himself as if he were centuries older than the rector. There is nothing in medical literature or practice that he does not know; and nothing at the bedside or in the out-patients' department that he cannot do. His seniors, especially if they be family doctors, he regards as the very stalest of "fogies," and their opinions as not worth a moment's consideration compared with his own assured knowledge. Now the average hearer knows when a curate is making a fool of himself in the pulpit, because he has some knowledge of the subject which the curate commonly talks about. But the young medical man may spout nonsense by the octavo, and the average patient has no means whatever of knowing whether he is talking like an Æsculapius or like a simpleton.

It may be said that the work of the young medical man is of less importance than that of the curate, inasmuch as the body is of less value than the soul. But that is not the way to compare the work of the two. The curate is never, at twenty-three, given the full charge and responsibility of a parish; he is merely a helper under the supervision and direction of an experienced senior. The youthful doctor of twenty-one may be in full charge of a whole practice, without supervision or direction of any sort. No one, surely, will contend that the power of life and death which he is called upon to exercise daily is a less important responsibility than that of reading the service in church and preaching a quarter of an hour's sermon two or three times a-month. The responsibility of the doctor in full charge is, in fact, quite immeasurably greater than that of the ordinary curate.

Some readers may, perhaps, be startled by the very considerable changes we are about to propose. But we are among those who believe that the scientific way of life is but just beginning; and that when the temper of science becomes generally prevalent very marked changes will be made in many directions, and that with a promptitude quite unfamiliar to our forefathers. Our suggestions are that no young man shall in future be allowed to take any sort of medical diploma or degree before he is twenty-three years of age; and that no man, however many and high the degrees he may have taken, shall receive a licence to practise independently, and on his own account, until he is twenty-five. Very few members of the general public will disagree with this. Ordinary persons show their appreciation of age in medical men by fighting shy of any practitioner who is not well on his way to forty. An outcry may be made against any change in the direction of later licensing by a certain number of medical parents and by a good many students. But the ideas of senior medical students and of very young practitioners on these points are of comparatively small importance. No doubt such persons can make a good deal of noise, and can influence their parents considerably against further changes; but the value of their judgment is, for the most part, inversely proportionate to the loudness of their talk.

The General Medical Council has decided that in future young men shall devote five years to medical study, instead of four and three as heretofore. If our suggestions were adopted, and no man were allowed to obtain a degree until he was twenty-three years of age, there would be no inducement to any raw schoolboy to begin medical study before he was eighteen. That would be all clear gain, because he would then be able to acquire something more than the mere rudiments of a general education. The general education of large numbers of medical practitioners is incredibly meagre. We do not say these things to shame or disparage our medical confrères, but to insist upon an improved state of things. General culture is a tree that grows more rapidly than any other in our fast moving times; and the doctor will by no means be able to hold the position he has gained, and which it is desirable he should have, if his general education be not superior to that of the average Board school master. Besides, the medicine of modern times cannot be comprehended at all by an untrained mind. The old fashioned practitioner is as much out of date as one of Queen Anne's coach horses would be if paired with a railway engine. It is often said, by way of a last argument for early licensing and a short term of study, that change in the direction of longer study and later licensing will altogether exclude poor men from the ranks of medicine. That we do not believe. The poor man still finds his way, through university scholarships and by other means, to the church and the bar. There is no doubt he would find his way into medicine, even if the entrance were three times as difficult as it now is. But if not, it is better to give facilities to the poor man in the shape of additional scholarships than to lower the level of general acquirements below that of the average culture of the times. If doctors, we repeat, are to hold their own with educated men they must themselves be educated; and as those who are already in the medical profession are the natural guardians of its honour and its efficiency, they must insist that the new entrants at the medical portals shall be at least abreast of the advancing standards of the times. We commend again both to medical and general readers the two suggestions, that no young man shall be allowed to obtain a medical qualification until he has reached the age of twenty-three, and that no person shall be licensed to practice independently and on his own account until he is twenty-five.

